# Supplementary material for: Modeling genetic epileptic encephalopathies using brain organoids
Source: EMBO Mol Med. 2021 Jul 15;13(8):e13610. doi: 10.15252/emmm.202013610 (PMC8350905; doi:10.15252/emmm.202013610)
Supplement: Supplementary file 1 — Appendix [file EMMM-13-e13610-s004.docx]

Modeling Genetic Epileptic Encephalopathies using Brain Organoids

**Table of contents:**

Appendix Table S1: List of exact p-values presented in the manuscript. Page 2-3

**Appendix Table S1**

**List of exact p-values presented in the manuscript.**

| **Figure** | **Description** | **Exact P-value** | **Test used** |
| --- | --- | --- | --- |
| **Appendix Fig S2A** | Week 6: WT vs. KO | 0,3675 | Ordinary one-way ANOVA with Tukey's multiple comparisons test |
|  | Week 6: WT vs. W-AAV | 0,9725 |  |
|  | Week 6: KO vs. W-AAV | 0,7857 |  |
|  | Week 10: WT vs. KO | 0,5619 | Ordinary one-way ANOVA with Tukey's multiple comparisons test |
|  | Week 10: WT vs. W-AAV | 0,4732 |  |
|  | Week 10: KO vs. W-AAV | 0,1368 |  |
|  | Week 15: WT vs. KO | 0,9949 | Ordinary one-way ANOVA with Tukey's multiple comparisons test |
|  | Week 15: WT vs. W-AAV | 0,8981 |  |
|  | Week 15: KO vs. W-AAV | 0,9291 |  |
|  | Week 18: WT vs. KO | 0,8952 | Ordinary one-way ANOVA with Tukey's multiple comparisons test |
|  | Week 18: WT vs. W-AAV | 0,9775 |  |
|  | Week 18: KO vs. W-AAV | 0,9828 |  |
|  |  |  |  |
| **Appendix Fig S3C** | Distribution of the mean for dorsal genes: KO vs WT | 1 | Kruskal-Wallis test |
|  | Distribution of the mean for ventral genes: KO vs WT | 0,064 |  |
|  | Distribution of the combined mean for dorsal and ventral genes | 1 |  |
|  |  |  |  |
| **Appendix Fig S4E** | WSM F1 vs WSM M2 | 0,0952 | Two-tailed unpaired Welch’s t-test |
|  |  |  |  |
| **Appendix Fig S5D** | GFAP: WSM F1 vs WSM S5 | 0,0311 | Two-tailed unpaired Welch’s t-test |
|  | S100B: WSM F1 vs WSM S5 | 0,0237 |  |
|  | AQP4: WSM F1 vs WSM S5 | 0,0077 |  |
|  | ALDH1A1: WSM F1 vs WSM S5 | 0,0061 |  |
|  |  |  |  |
| **Appendix Fig S5F** | γH2AX: WSM F1 vs WSM S5 | 0,0025 | Two-tailed unpaired Welch’s t-test |
|  | 53BP1: WSM F1 vs WSM S5 | 0,0292 |  |
|  |  |  |  |
| **Appendix Fig S5H** | WNT1: WSM F1 vs WSM S5 | 0,5394 | Two-tailed unpaired Welch’s t-test |
|  | WNT2B: WSM F1 vs WSM S5 | 0,045 |  |
|  | WNT3: WSM F1 vs WSM S5 | 0,8571 |  |
|  | WNT3A: WSM F1 vs WSM S5 | 0,8065 |  |
|  | WNT5A: WSM F1 vs WSM S5 | 0,0032 |  |
|  | WNT8B: WSM F1 vs WSM S5 | 0,271 |  |
|  | ROR2: WSM F1 vs WSM S5 | 0,5449 |  |
|  | AXIN2: WSM F1 vs WSM S5 | 0,0448 |  |
|  | CCND1: WSM F1 vs WSM S5 | 0,6862 |  |
|  | LEF1: WSM F1 vs WSM S5 | 0,8573 |  |
|  | TCF7: WSM F1 vs WSM S5 | 0,0306 |  |
|  | TCF3: WSM F1 vs WSM S5 | 0,0404 |  |
|  | TCF4: WSM F1 vs WSM S5 | 0,1218 |  |
|  | CTNNB1: WSM F1 vs WSM S5 | 0,0169 |  |
|  |  |  |  |
| **Appendix Fig S5I** | TBR1: WSM F1 vs WSM S5 | 0,1736 | Two-tailed unpaired Welch’s t-test |
|  | BCL11B: WSM F1 vs WSM S5 | 0,0467 |  |
|  | SATB2: WSM F1 vs WSM S5 | 0,4278 |  |
|  | POU3F2: WSM F1 vs WSM S5 | 0,1315 |  |
|  | CUX1: WSM F1 vs WSM S5 | 0,0074 |  |
|  | RELN: WSM F1 vs WSM S5 | 0,1423 |  |
