# Supplementary material for: Modeling genetic epileptic encephalopathies using brain organoids
Source: EMBO Mol Med. 2021 Jul 15;13(8):e13610. doi: 10.15252/emmm.202013610 (PMC8350905; doi:10.15252/emmm.202013610)
Supplement: Supplementary file 2 — Expanded View Figures PDF [file EMMM-13-e13610-s001.pdf]

Expanded View Figures

Figure EV1. Genetic editing of hESCs and generation of WWOX-KO COs.

A Western blot (WB) analysis of WiBR3 hESC individual colonies after CRISPR-editing targeted to exon 1 of the WWOX gene. Clone 1B (KO1) and clone A2 (KO2) were selected for the organoid generation. MCF-7 was used as a positive control that highly expresses WWOX.
B WB analysis of week 6 COs for validation of WWOX expression levels.
C Sanger sequencing of the WWOX-KO clones. The vertical line specifies the end of exon 1 and the start of the adjacent intron, the blue line indicates the nucleotides inserted following the genetic editing, and the black horizontal line underlines the premature termination codon (PTC) resulting from the frame shift.
D WWOX-KO WiBR3 hESCs were introduced with a plasmid containing WWOX coding sequence and targeting the safe harbor locus AAVS1. This resulted in hESCs overexpressing WWOX under UBP promoter from the AAVS1 locus (W-AAV), as visualized in this WB analysis of KO1 hESC clones introduced with WWOX coding sequence.
E Week 24 COs stained for SOX2, β3-tubulin and WWOX. Arrowhead denotes a $SOX2^+$ cell that expresses WWOX, and arrow denotes $SOX2^-$ cell that expresses WWOX. (WT: *n* = 10 from 4 batches, KO: *n* = 9 from 3 batches). Scale = 50 μm (left), 15 μm (right).
F Top panel: Schematic of organoid slice setup. A borosilicate glass electrode is used for local field potential recordings (LFP) and is positioned 150 μm from the edge of the slice (indicated by white bar). A sample recording is shown (a) before and (b) after administration of 100 μM 4-AP. Bottom panel: Flowchart of steps used for signal processing field recordings. (IIR = infinite impulse response, FFT = fast Fourier transform, AUC = area under the curve).
G Mean spectral power of week 7 WT and KO COs in baseline conditions. The line marks the low gamma range (30–79.9 Hz). Statistical significance was determined using the two-tailed unpaired Welch's *t*-test (WT: *n* = 14 slices, 5 organoids, and 3 batches; and KO: *n* = 14 slices, 8 organoids, 3 batches).
H Normalized area under the curve of the mean spectral power in (G) for the low gamma range (30–79.9 Hz). Data represented by mean ± SEM. The two-tailed unpaired Student's *t*-test was used to test statistical significance. The numerals in all bars indicate the number of analyzed slices and organoids (i.e., slices (organoids)).
I Normalized area under the curve of the mean spectral power WT and 2 KO lines at week 7 and 15 in baseline conditions, for the 0.25- to 1-Hz frequency range. The two-tailed unpaired Welch's *t*-test was used to test statistical significance. The numerals in all bars indicate the number of analyzed slices and organoids (i.e., slices (organoids)).
J Normalized area under the curve of the mean spectral power of WT and KO lines at week 7 in baseline and 100 μM 4-AP conditions, for the 0.25- to 1-Hz frequency range. The two-tailed unpaired Welch's *t*-test was used to test statistical significance. The numerals in all bars indicate the number of analyzed slices and organoids.

Data information: n.s (non-significant), $^*P \leq 0.05$, $^{**}P \leq 0.01$.

© 2021 The Authors

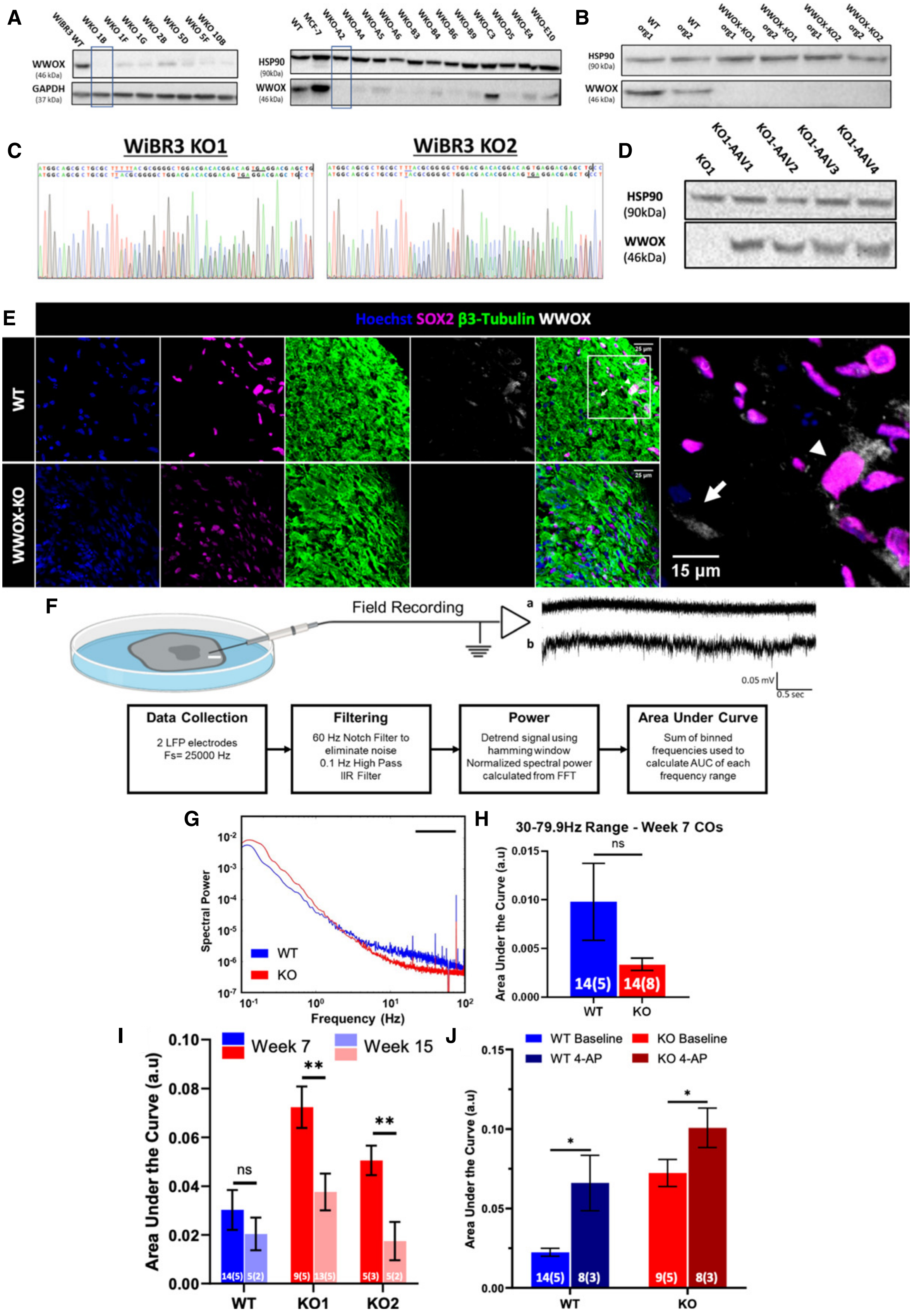

Figure EV1.

 © 2021 The Authors

Figure EV2. WWOX-KO cerebral organoids showed enhanced astrogenesis and loss of checkpoint inhibition.

A Western blot analysis of week 24 COs, examining the protein expression of WWOX, GFAP (astrocytes), SOX2 (RG cells), and NeuN (mature neurons).

B A summary of the band intensities of the Western blot exemplified in (A), presented as mean ± SEM of the WT COs and KO COs, and as fold change compared with the WT COs (WT: *n* = 2 from 1 batch; KO: *n* = 4 from 1 batch).

C IF staining of week 10 COs with the proliferation marker Ki67 localized with S100β (astrocytes) and SOX2 (RGs) (WT: *n* = 6 from 2 batches; KO: *n* = 4 from 2 batches).

D Quantification of (C). The boxplot represents the 1st and 3rd quartile, with its whiskers showing the minimum and maximum points and a central band representing the median. Statistical significance was determined using the two-tailed unpaired Welch's *t*-test (WT: *n* = 6 from 2 batches; KO: *n* = 4 from 2 batches).

E Week 10 COs stained for Ki67 (proliferation), γH2AX (DNA breaks) and SOX2 (RGs) (WT: *n* = 6 from 2 batches; KO: *n* = 4 from 2 batches).

F Quantification of γH2AX+/Ki67+ double-positive cells (DPCs) normalized to the number of SOX2+ cells, corresponding to the size of the VZ, seen in (C). The boxplot represents the 1st and 3rd quartile, with its whiskers showing the minimum and maximum points and a central band representing the median. Statistical significance was determined using the two-tailed unpaired Welch's *t*-test (WT: *n* = 6 from 2 batches; KO: *n* = 4 from 2 batches).

G Week 10 COs co-stained for the apoptosis marker cleaved caspase-3 and the RG marker SOX2 in the VZ of WT, KO and W-AAV COs (WT: *n* = 6 from 2 batches; KO: *n* = 4 from 2 batches; and W-AAV: *n* = 4 from 1 batch). Scale = 50 μm.

H Quantification of the data presented in (G). The boxplot represents the 1st and 3rd quartile, with its whiskers showing the minimum and maximum points and a central band representing the median. Statistical significance was determined using one-way ANOVA with Tukey's multiple comparisons test (WT: *n* = 6 from 2 batches; KO: *n* = 4 from 2 batches; W-AAV: *n* = 4 from 1 batch).

Data information: n.s (non-significant), $^{*}P \leq 0.05$, $^{**}P \leq 0.01$.

© 2021 The Authors

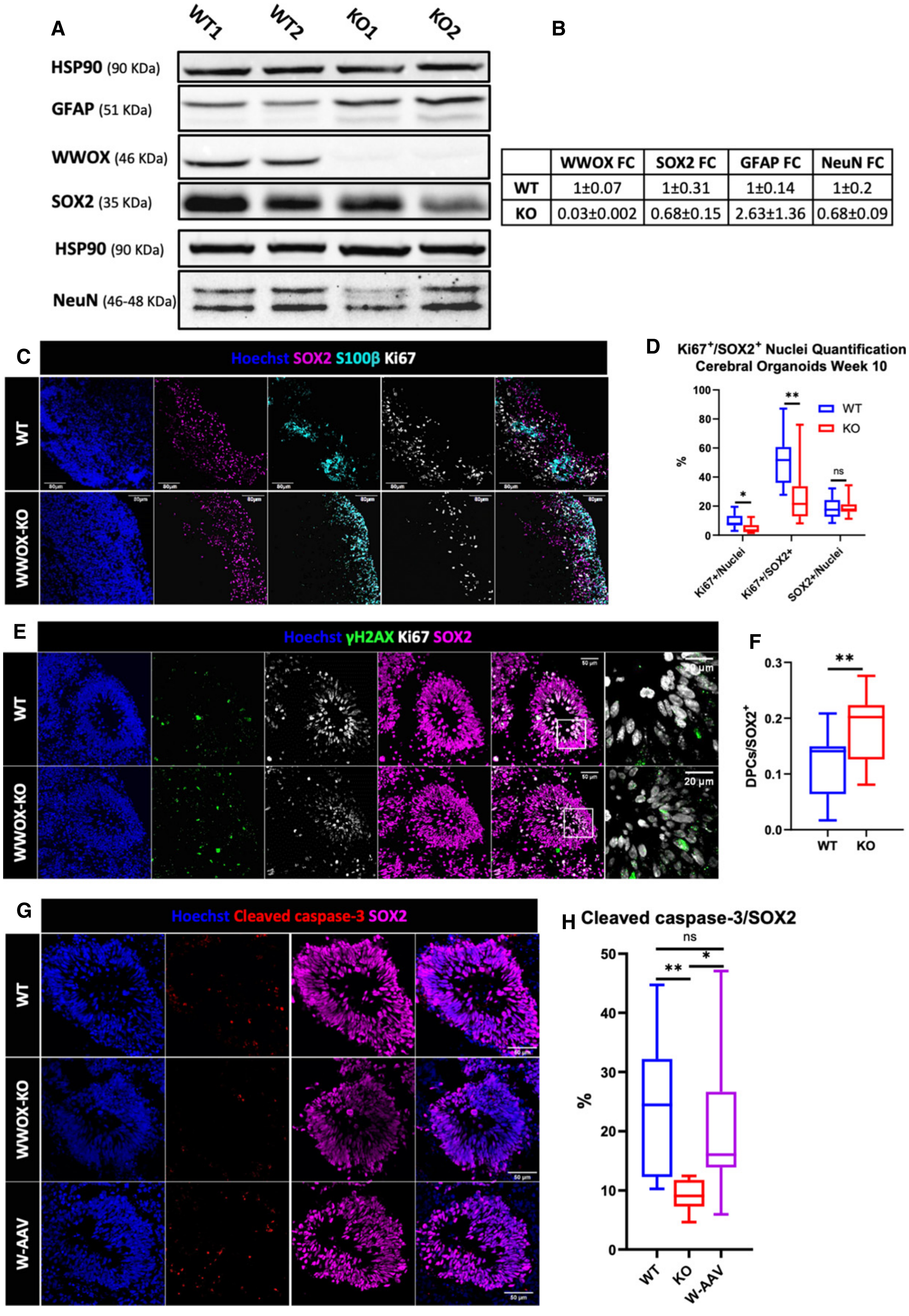

	WWOX FC	SOX2 FC	GFAP FC	NeuN FC
WT	1±0.07	1±0.31	1±0.14	1±0.2
KO	0.03±0.002	0.68±0.15	2.63±1.36	0.68±0.09

Figure EV2.

© 2021 The Authors

Figure EV3. Cerebral organoid RNA sequencing revealed major differentiation defects.

A Principal component analysis (PCA) plot using the top two principal components, showing the RNA-seq data form week 15 COs. PCA revealed two distinct clusters corresponding to the biological identity of the sample—WT or WWOX-KO.

B Volcano plot representing differentially expressed genes in the sequenced COs based on fold change (x-axis) and *P*-value (y-axis). The *P*-value was calculated using the Wald test.

C qPCR analysis of the kinetics of Wnt-related genes at week 6, 10, 15 and 24 COs. Data are represented as mean ± SEM. Statistical significance was determined using the two-tailed unpaired Welch's *t*-test (WT W6: $n = 3$; KO W6: $n = 3$; WT W10: $n = 3$; KO W10: $n = 3$; WT W15: $n = 4$; KO W15: $n = 4$; WT W24: $n = 4$; and KO W24: $n = 3$).

D qPCR for markers of the six different layers of the human cortex in week 15 COs. Data are represented as mean ± SEM. Statistical significance was determined using one-way ANOVA with Tukey's multiple comparisons test (WT: $n = 4$ from 1 batch; KO: $n = 4$ from 1 batch; and W-AAV: $n = 4$ from 1 batch).

E Immunofluorescent staining of cortical layer markers SATB2, CTIP2, and TBR1 in week 24 COs (WT: $n = 10$ from 4 batches; KO: $n = 9$ from 3 batches; and W-AAV: $n = 4$ from 1 batch).

F Quantification of the cortical markers seen in E, normalized to the total number of nuclei. The y-axis indicates the fold change compared with the average of the WT COs. The boxplot represents the 1st and 3rd quartile, with its whiskers showing the minimum and maximum points and a central band representing the median. Statistical significance was determined using one-way ANOVA with Tukey's multiple comparisons test (WT: $n = 10$ from 4 batches; KO: $n = 9$ from 3 batches; and W-AAV: $n = 4$ from 1 batch batches).

Data information: *$P \le 0.05$, **$P \le 0.01$, and ***$P \le 0.001$.

© 2021 The Authors

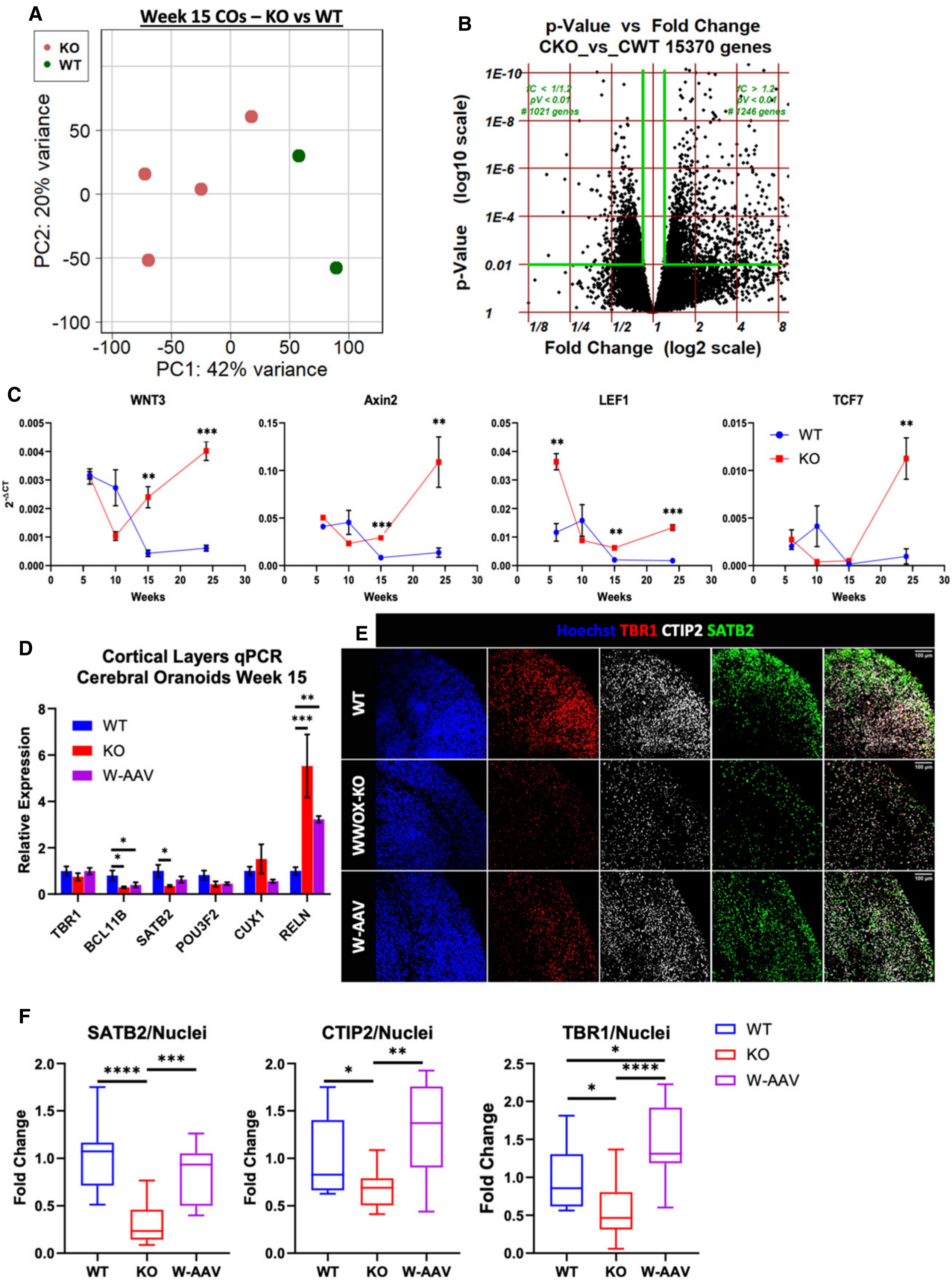

Figure EV3.

 © 2021 The Authors

Figure EV4. Spinocerebellar ataxia type 12 forebrain organoids presented normal cortical development.

PBMCs were isolated from SCAR12 (G372R mutation) patients and from their healthy parents were reprogrammed into iPSCs, and subsequently were differentiated into forebrain organoids.

A Week 4 forebrain organoids (FOs) of the healthy, heterozygote father (WPM F2) and mother (WPM M3), and their sick homozygotes daughter (WPM D1) and son (WPM S1) stained for WWOX, β3-tubulin, and SOX2 expression (WPM F2: $n = 2$ from 1 batch; WPM M3: $n = 2$ from 1 batch; WPM D1: $n = 2$ from 1 batch; and WPM S1: $n = 2$ from 1 batch). Scale = 50 μm (left), 20 μm (right).

B qPCR for the assessment of the expression of different neural markers in week 20 FOs.

C qPCR for the measurement of expression levels of cortical layer markers in week 20 FOs.

D Expression level of Wnt pathway-related genes at mRNA levels quantified using qPCR in week 20 FOs.

Data information: B-D: Data are represented as mean ± SEM. Statistical significance was determined using one-way ANOVA with Tukey's multiple comparisons test (WPM F2: $n = 4$ from 1 batch; WPM M3: $n = 3$ from 1 batch; WPM D1: $n = 4$ from 1 batch; and WPM S1: $n = 3$ from 1 batch). $*P \leq 0.05$, $**P \leq 0.01$, $***P \leq 0.001$, and $****P \leq 0.0001$.

© 2021 The Authors

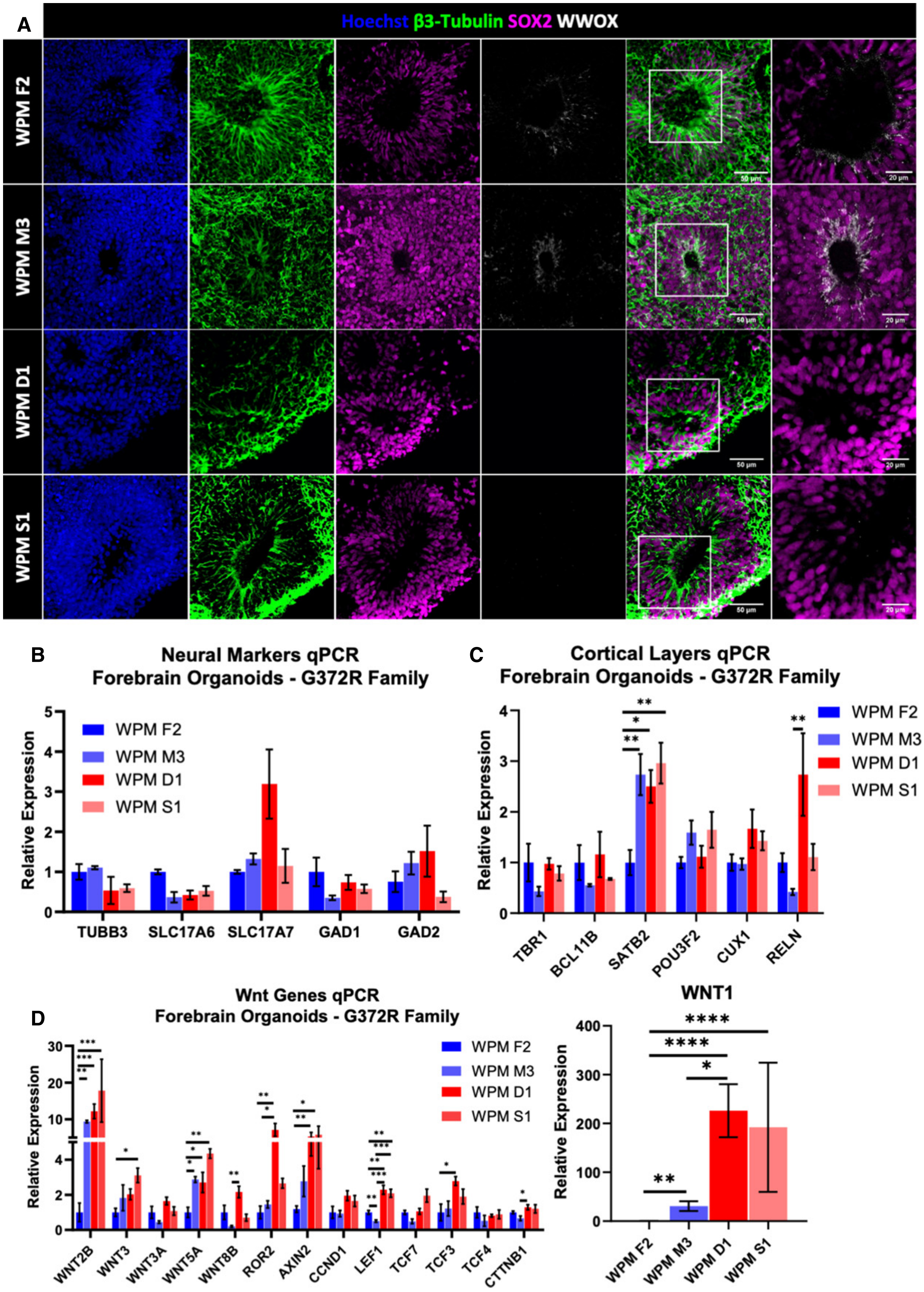

Figure EV4.

 © 2021 The Authors

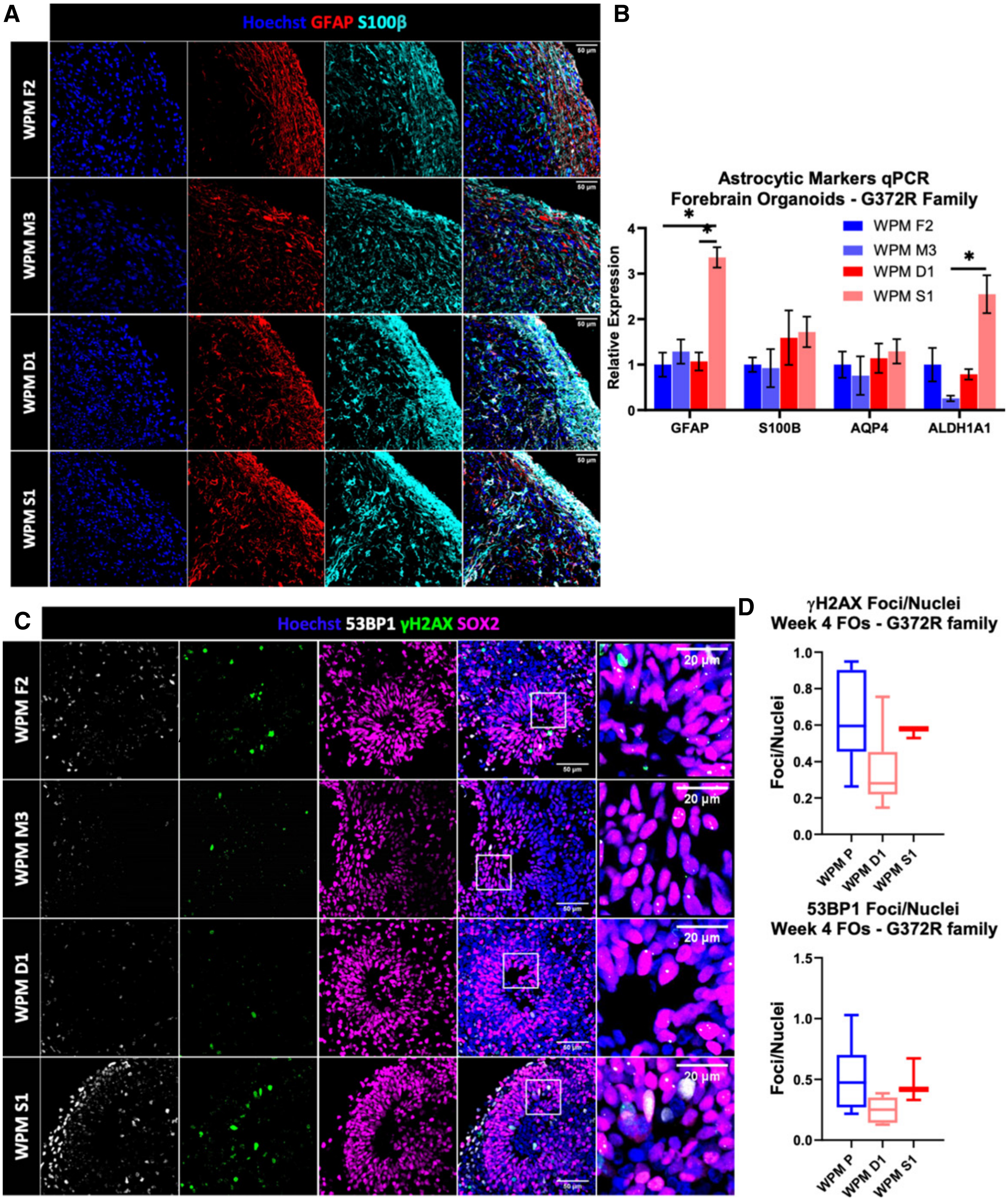

Figure EV5. Spinocerebellar ataxia type 12 forebrain organoids presented normal astrogenesis and DNA damage.

A Week 20 FOs stained for the astrocytic markers GFAP and S100β, showing comparable levels of expression in week 20 WPM D1 and WPM S1 FOs compared with the age-matched WPM F2 and WPM M3 FOs (WPM F2: n = 3 from 1 batch; WPM M3: n = 3 from 1 batch; WPM D1: n = 3 from 1 batch; and WPM S1: n = 3 from 1 batch). Scale = 50 μm.

B qPCR quantifying the transcript levels of astrocytic markers in week 20 FOs. Data are represented as mean ± SEM. Statistical significance was determined using one-way ANOVA with Tukey's multiple comparisons test (WPM F2: n = 4 from 1 batch; WPM M3: n = 3 from 1 batch; WPM D1: n = 4 from 1 batch; and WPM S1: n = 3 from 1 batch). *$P \le 0.05$.

C Week 4 FOs stained for the DNA damage markers γH2AX and 53BP1, together with the RG marker SOX2. Comparison of the foci demonstrated no significant difference in sites of damage in the nuclei of cells in the VZ at physiological conditions (WPM F2: n = 1; WPM M3: n = 2; WPM D1 n = 2; and WPM S1: n = 2). Scale = 50 μm (left), 20 μm (right).

D Quantification of the staining presented in figure C. The boxplot represents the 1st and 3rd quartile, with its whiskers showing the minimum and maximum points and a central band representing the median. Statistical significance was determined using one-way ANOVA with Tukey's multiple comparisons test (WPM P: n = 3; WPM D1: n = 2; and WPM S1: n = 2). WPM P = WPM F2 and WPM M3, both are healthy heterozygotes, grouped together.
